# Role of dewatering and roasting parameters in the quality of handmade gari

**DOI:** 10.1111/ijfs.14745

**Published:** 2020-09-05

**Authors:** Layal Dahdouh, Andrés Escobar, Eric Rondet, Julien Ricci, Geneviève Fliedel, Laurent Adinsi, Dominique Dufour, Bernard Cuq, Michèle Delalonde

**Affiliations:** ^1^ CIRAD UMR QualiSud Montpellier F‐34398 France; ^2^ QualiSud Univ Montpellier CIRAD Montpellier SupAgro Univ Avigon Univ La Réunion Montpellier France; ^3^ CIAT CGIAR Research Program on Roots Tubers and Bananas Km 17 via Cali‐Palmira Cali AA6713 Colombia; ^4^ Agronomic Sciences Faculty University of Abomey‐Calavi Cotonou 01 BP 526 Benin

**Keywords:** Cassava, gari, mash water content, quality attributes, roasting parameters

## Abstract

Gari is a common cassava precooked dried semolina in sub‐Saharan Africa. Our study investigated the role of process parameters and mash water content on gari quality during traditional roasting stage. The statistical analysis for eight quality criteria revealed that gari quality is highly influenced by the process parameters adopted by the operators. To emphasise the twin impact of roasting parameters and mash water content, different mashes with varying water content were roasted leading to different adjustments of the roasting conditions according to the operators. When the variability of the water content becomes greater, a greater variability in the final quality of the obtained garis was observed between operators (lightness, swelling capacity, starch content, texture and colour). These results suggest that technological improvements to the gari process could be achieved by appropriate management of the roasting and dewatering parameters.

## Graphical abstract

Role of process parameters in gari quality during handmade roasting of mashes with different water content.

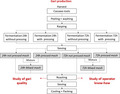

## Introduction

Cassava (*Manihot esculenta* Crantz) roots are the raw material for gari production. Gari consists of granulated cassava particles, which have been fermented to a lesser or greater degree depending on regional preferences (Adinsi *et al*., 2019) and then partially gelatinised by roasting. It is very widely consumed in West Africa due to its high energy value, affordable price, long shelf‐life and ease of use in or with many local dishes (Maduagwu & Fafunso, 1980; Oduro *et al*., 2000; Akosua & Bani, 2007). The quality of gari can differ from one processing operator to another, and sometimes from one batch to another (Akosua & Bani, 2007).

Quality attributes can be assessed by standards stipulated by the Codex Alimentarius or by national standards (Codex Alimentarius Commission, 1995), as well as by consumer preferences, which vary among gender (Teeken *et al*., 2018) and also among countries or regions. For example, consumers in rural areas are generally less demanding in terms of nutritional and sanitary quality than city dwellers. Gari quality attributes also depend on consumption patterns, for example cooked in boiling water, eaten dry as a snack or cooked into a paste (Ezedinma & Moses, 2008; Arisa *et al*., 2011; Makanjuola *et al*., 2012) and on sensory characteristics and acceptance criteria (Bechoff *et al*., 2016).

At the artisanal level, gari is generally processed in the open air, on premises and with equipment lacking health and safety controls, as well as potable water (Codex Alimentarius Commission, 1995). The process involves numerous unit operations, such as peeling, washing, rasping, fermentation, pressing, sieving and roasting (simultaneously a cooking and drying operation; Akinoso & Olatunde, 2014). These unit operations are labour‐intensive; hence men, women and children are typically involved in the manufacturing process. Children are involved in peeling and cleaning the roots, men mainly in the rasping and pressing operations, while women, often accompanied by infants or very young children, roast the product under highly precarious conditions, without protection either from the smoke or heat released by the wood fire. In addition to roasting which is really a tedious operation (Sobowale *et al*., 2017), some operations such as washing and pressing generate nauseating and toxic effluents due to the presence of cyanide released from the roots. These starch‐rich effluents are not collected or managed and thereby also promote bacterial development. While cassava processing into gari constitutes an essential source of income and nutrition, it can also pose major health risks to the local populations.

The objective of this work was to study the impact on gari quality of process parameters adopted by the operators during the roasting stage of various mashes with varying water content, with a view towards recommending technological improvements to this operation. These improvements are aimed both at protecting operators from risks associated with this operation, and at ensuring that the gari is compliant in terms of the major quality attributes. The manner in which roasting is done strongly impacts gari acceptability and therefore on its sale price (Makanjuola *et al*., 2012). In order to meet the above objective, products obtained from the fermentation/pressing/sieving operations either performed conventionally, or conversely deliberately non‐optimally, were roasted in different conditions depending on the operators. This approach ensured controlled variability of the fermented mash characteristics. The results should help the operators to adapt roasting parameters according to the water content of the mash in order to ensure a safe and good quality of gari.

## Materials and methods

### Overview

The experimental approach consisted in (i) providing two experienced gari roasting operators with four sample types to be roasted: cassava mash that had been fermented for 24 and 72 h, and after each of those periods, prepared with both a normal and a higher‐than‐normal water content; (ii) analysing operators’ behaviour in terms of nine process parameters: mash quantity, number of additions during roasting, mass of additions, intermediate roasting times, wok temperature upon an addition, roasting product water content before each addition, total roasting time, total stirring time and duration of the various stirring cycles; (iii) studying the characteristics of the gari obtained via eight roasted gari quality criteria. Among these criteria, cyanide potential and water content stand out as major health and safety quality attributes. Eight other criteria were adopted for analysing the roasted gari: median particle size (d_50_), colour, fibre content, titratable acidity, pH, starch content, starch gelatinisation rate and gari swelling capacity. These quality criteria were selected based on literature (Codex Alimentarius Commission, 1995; Makanjuola *et al*., 2012; Escobar *et al*., 2018) and on local surveys in relation with consumer acceptance. As for responses characterising the operators’ behaviour and hence the process parameters, they were partially selected based on literature (Ezeocha *et al*., 2019) and local survey but mostly after a meticulous analysis of the video recordings during roasting step.

### Raw materials and gari production under local conditions

Cassava processing into gari was carried out in a small processing unit located in Ikpinlé in Benin. Roots from the *Odoungbo* variety were harvested and processed within the following 24 h (freezing was avoided since it might induce some physico‐chemical changes (Oyeyinka *et al*., 2019).

**Figure 1:**
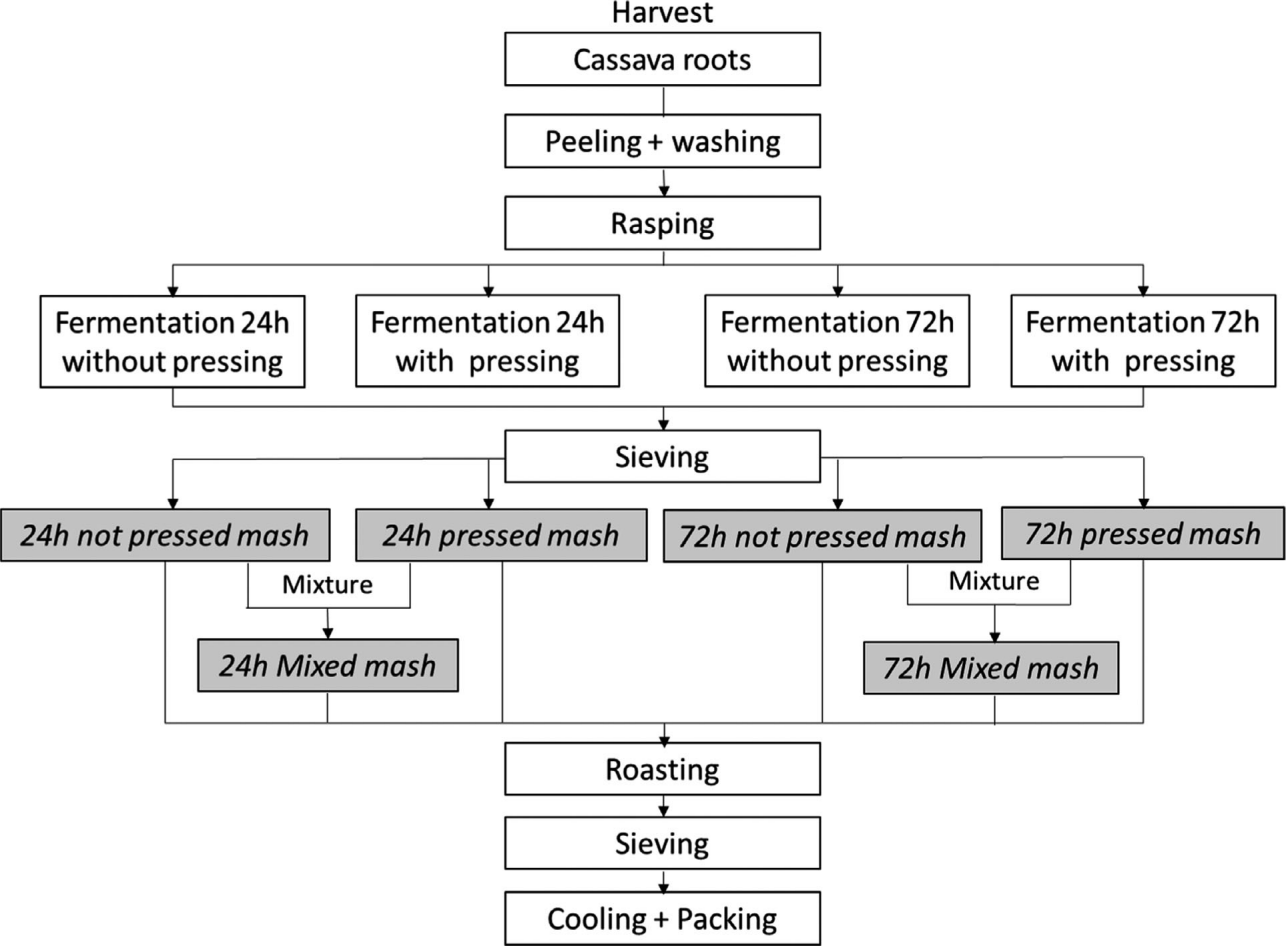
Description of cassava processing into six different garis derived from 6 intermediate preroasting products.

The processing into gari was conducted under local conditions, via the following main successive operations: harvest, peeling and washing, rasping, fermentation, pressing, sieving, roasting, cooling and packing (Fig. 1).

#### Harvest, peeling, washing and rasping

Immediately after harvest, the fresh cassava roots were washed in water. Roots were peeled using knives, which are used both to remove the peel and cut off the root tips. Then, the cassava roots were rasped using a motorised mechanical rasper running at 511 ***g***. The rasping drum was composed of cylindrical sheet metal, rolled around a wooden cylinder (diameter 19 cm). Fifty‐five per cent of the surface was perforated with 5 mm dia. holes to create a raised, abrasive surface.

#### Fermentation and pressing

The mash obtained after rasping was bagged at ambient temperature for defined periods of time to ferment, to develop the required organoleptic properties, and to reduce the mash water content to 40–50% (wb).

In order to generate fermented products with varied properties, four fermentation conditions were adopted (Fig. 1 and Table 1): two fermentation times (24 or 72 h) and non‐pressed or pressed conditions (the pressed condition is the traditional one).

**Table 1:** Nomenclature and dry matter of the 12 fermented mashes used during the roasting step by the two operators (W1 and W2)

Fermentation time	24 h		72 h	
Pressing	No	Yes	No	Yes
Nomenclature of fermented mash for operator 1 (W_1_) and 2 (W_2_)	W_1_ 24 h NP 46.5%*	W_1_ 24 h P 50.5%*	W_1_ 72 h NP 46.5%*	W_1_ 72 h P 54.6%*
	W_1_ 24 h M 48.7%*		W_1_ 72 h M 49.6%*	
	W_2_ 24 h NP 46.7%*	W_2_ 24 h P 54.3%*	W_2_ 72 h NP 47.3%*	W_2_ 72 h P 54.9%*
	W_2_ 24 h M 49.1%*		W_2_ 72 h M 49.0%*	

* Dry matter content.

In order to produce these four products (i) approximately 2x100 kg batches of mash were left to ferment for 24 h or 72 h in two different plastic bags under ambient conditions (35°C in the day, 25°C in the night) without pressure; and (ii) approximately 2 × 100 kg batches of mash were left to ferment for 24 h or 72 h in two other plastic bags in the same ambient conditions but under pressure. The pressing unit comprised a manually activated mechanical screw press.

#### Sieving

After fermentation, the mash (either compressed (P) or not (NP)) was broken by hand into small pieces, and the four different broken cakes were then sieved using a 3.35 mm mesh sieve.

Hence, four products were generated (24 h P, 24 h NP, 72 h P and 72 h NP; Table 1). With these four products, two additional products were generated: the mash batches after 24 h or 72 h fermentation were mixed 1:1 after sieving to give mixed mash (Table 1).

In total six products with different dry matter contents were obtained and divided into equal parts for roasting by both operators, thereby producing 12 samples for gari‐making (Table 1). Two experienced operators were selected for the roasting operation and designated operators 1 and 2, named W1 and W2, respectively.

#### Roasting

Gari was roasted using a metal wok placed on a wood fire (traditional cooking). The objective of roasting is to (i) gelatinise starch; (ii) dry the product to eliminate sufficient water to promote a long shelf‐life; and (iii) roast to confer specific sensory properties to the gari. This is a delicate operation which requires know‐how and skill to be performed successfully. It is also arduous in terms of the physical effort required by the equipment operation and discomfort caused by the smoke that the operator must withstand throughout roasting.

The dry matter of the 12 fermented mash samples was measured just before roasting. The operator progressively, in three to four steps, added between 2.2 and 4.5 kg at a time (depending on the operator) to the wok, with roasting between such additions. During roasting, the product was continually stirred by the operator using a triangular piece of gourd shell. The time between each addition varied depending on the operator. At the end of roasting, the gari was then collected in basins and left to cool.

### Physico‐chemical quality attributes of gari

The analysed product quality attributes were based on macroscopic (water content, particles size, colour) and microscopic (physico‐chemical) responses as described by Escobar *et al*. (2018).

#### Dry basis water content

Samples were dried in an oven (Binder Model ED 23 Series Gravity Convection Oven, BINDER Inc., Bohemia, USA) at 105°C until a constant mass was reached. The dry basis water content (*w*) of the different fermented mashes and garis was determined using Equation 1, where *m_W_* = the water mass evaporated during drying and *m_s_* = the mass of the solid after drying.

(1) $$\text{SG}\left(\%\right)=\frac{\left(\Delta H_{\text{pulp}}‐\Delta H_{\text{gari}}\right)}{\Delta H_{\text{pulp}}}\times 100$$

#### Particle size distribution

The particle size distribution of 100 g of gari was determined using a set of 7 sieves (200 mm diameter) (Test Sieve, Fisher Scientific Co., Portsmouth, USA) with a decreasing mesh size (3.35, 2.00, 1.40, 0.85, 0.60, 0.30 and 0.15 mm), and shaken mechanically (Ro‐Tap RX‐29‐E, W.S. Tyler, Ohio, USA) in *fine particles* mode, for 5 min. The median particle diameter (d_50_) was given for each analysis. All analyses were performed at least in duplicate.

#### Colour

Gari colour was determined in triplicate according to the L*a*b* colour notation system using a Minolta Chroma Meter (CR‐400 Konica Minolta, Japan). a* indicates the degree of redness or greenness, while L* and b* relate to the degree of lightness and the intensity of yellowness, respectively (Penfield & Campbell, 1990).

#### Cyanogenic potential

Three independent samples were collected from the mash (before fermentation) and from processed garis. The cyanide released (HCN) was quantified using the colorimetric procedure (Essers *et al*., 1993; Sánchez *et al*., 2009) and expressed as µg HCN g^‐1^ dry matter according to Escobar *et al*. (2018).

#### Titratable acidity

Titratable acidity of gari was measured in triplicate using an automatic Titroline apparatus (Schott Schweiz AG, St. Gallen, Switzerland). Titratable acidity (g of lactic acid/100 g of sample, dry weight) was evaluated by titration with 0.1 N NaOH to reach the equilibrium pH of 8.3 according to Escobar *et al*. (2018).

#### Fibre content

Total dietary fibre content (TDF) was measured on dried gari using an enzyme kit (K‐TDFR, Megazyme International, Bray, Ireland). Total dietary fibre (TDF, % dry basis) was determined on triplicate samples.

#### Total starch content

Total starch content (S_T_C) was estimated with the enzymatic method developed by Holm *et al*. (1986) and Giraldo Toro *et al*. (2015). Total starch content (S_T_C, % dry basis) was determined on triplicate samples of dried material (garis).

#### Extent of gari starch gelatinisation

The enthalpy change (∆*H*, J g^−1^ of dry starch) caused by gelatinisation was measured by differential scanning calorimetry (DSC 8500 Perkin Elmer, Norwalk, USA) running at 10°C min^−1^, using a water/sample ratio of 5/1. The enthalpy of gelatinisation was determined on raw flour of cassava mash and gari. The extent of starch gelatinisation of gari (*SG* %) was calculated using Equation 2. Experiments were conducted in triplicate.

(2) $$\text{SG}\left(\%\right)=\frac{\left(\Delta H_{\text{pulp}}‐\Delta H_{\text{gari}}\right)}{\Delta H_{\text{pulp}}}\times 100$$

#### Gari swelling capacity

The gari swelling capacity was estimated in triplicate by measuring the volume by which a gari sample (10 mL) swelled after the addition of 40 mL of water, according to Ajibola *et al*. (1987), Olayinka Sanni (1994), Escobar *et al*. (2018).

Results were analysed by principal component analysis and correlation matrices.

### Statistical analysis

Multivariate analysis was carried out using XLSTAT (Addinsoft, New York, USA). PCA (principal component analysis) was performed to analyse the total variability between the different garis obtained by the two operators. Pearson’s correlation matrix was employed to explore the relationships among the different gari quality attributes.

### Analysis of the role of the process parameters during the roasting stage

The process parameters analysis was based on video recordings to describe the actions of the operators, along with additional measurements.

The video recordings were made throughout roasting for each of the products fermented for 24 h (pressed, non‐pressed, mixed), and for both operators. The analysis of these recordings was used to describe the stirring profile throughout the various stirring cycles. These parameters expressed the individual actions of each operator. Total roasting time and total stirring time were also recorded.

Six additional parameters were measured: (i) total mash mass per roasting operation (mash quantity); (ii) number of mash additions; (iii) mass of additions; (iv) individual roasting time for each addition; (v) wok temperature upon the additions (measured using a temperature sensor in the centre of the wok and connected to an ALMEMO (ALMEMO 2690‐8A; AHLBORN, Munich, Germany); and (vi) dry matter content of roasting product in the wok just prior to the additions.

## Results

### Physico‐chemical quality attributes of gari

Table 2 presents the physico‐chemical quality attributes of the different garis produced under different process conditions adopted by operators 1 and 2.

**Table 2:** Physico‐chemical quality attributes of the different garis produced by operators 1 and 2 (for legend, see Table 1)

Treatment	Swelling capacity (/)	pH (/)	Titratable acidity (%)	Colour			Total dietary fibre (mg/100 g d.b.)	Starch content (%)	Starch gelatinisation (%)	Dry matter (%)	Cyanide potential (µgHCN g^‐1^ dry matter)	Particle size d_50_ (mm)
				L* (/)	a* (/)	b* (/)						
W1 24 h P	3.0 (±0.1)	4.5 (±0.1)	0.6 (±0.1)	92.9 (±0.4)	‐0.8 (±0.1)	11.1 (±0.4)	21.4 (±1.6)	86.7 (±0.5)	81.8 (±1.4)	93.9 (±0.2)	15.8 (±0.1)	0.4 (±0.1)
W1 24 h NP	3.0 (±0.1)	4.7 (±0.1)	0.8 (±0.1)	93.6 (±0.1)	‐0.1 (±0.1)	9.6 (±0.1)	25.3 (±0.4)	86.7 (±0.2)	66.9 (±0.1)	93.1 (±0.6)	17.1 (±0.1)	0.6 (±0.1)
W1 24 h M	3.3 (±0.1)	4.6 (±0.1)	0.8 (±0.1)	91.5 (±0.1)	‐0.4 (±0.1)	11.6 (±0.2)	25.3 (±0.9)	84.4 (±0.4)	74.5 (±1.7)	93.1 (±0.1)	13.9 (±0.2)	0.5 (±0.1)
W1 72 h P	3.1 (±0.1)	4.1 (±0.1)	1.1 (±0.1)	91.5 (±7.9)	‐0.3 (±0.1)	10.7 (±0.1)	20.3 (±2.6)	85.9 (±0.6)	89.4 (±0.9)	95.1 (±0.1)	12.8 (±0.1)	0.4 (±0.1)
W1 72 h NP	2.8 (±0.1)	4.1 (±0.1)	1.5 (±0.1)	91.9 (±0.2)	0.2 (±0.1)	11.5 (±0.6)	21.0 (±1.8)	85.9 (±0.3)	70.7 (±1.4)	92.3 (±0.6)	13.9 (±0.1)	0.7 (±0.1)
W1 72 h M	3.0 (±0.1)	4.1 (±0.1)	1.4 (±0.1)	89.5 (±0.2)	0.9 (±0.1)	12.3 (±0.3)	21.7 (±0.6)	86.6 (±0.8)	80.6 (±0.1)	95.6 (±0.1)	19.8 (±0.3)	0.5 (±0.1)
W2 24 h P	3.0 (±0.1)	4.5 (±0.1)	0.6 (±0.1)	92.6 (±0.1)	‐0.1 (±0.1)	11.7 (±0.2)	21.5 (±0.8)	87.3 (±0.6)	87.2 (±0.5)	93.8 (±0.1)	4.4 (±0.1)	0.4 (±0.1)
W2 24 h NP	2.6 (±0.1)	4.5 (±0.1)	0.9 (±0.1)	81.8 (±0.1)	1.6 (±0.1)	16.3 (±0.8)	25.4 (±0.6)	79.6 (±0.7)	93.4 (±0.6)	88.7 (±0.3)	11.6 (±0.3)	1.1 (±0.3)
W2 24 h M	2.9 (±0.1)	4.5 (±0.1)	0.8 (±0.1)	87.2 (±0.2)	0.5 (±0.1)	15.5 (±0.3)	21.6 (±0.9)	80.5 (±1.5)	90.6 (±0.9)	92.2 (±0.1)	8.0 (±0.1)	0.7 (±0.2)
W2 72 h P	3.0 (±0.1)	3.9 (±0.3)	1.1 (±0.1)	93.3 (±0.1)	0.4 (±0.1)	10.2 (±0.2)	18.9 (±0.5)	87.7 (±0.2)	87.2 (±0.3)	94.1 (±0.1)	6.8 (±0.1)	0.4 (±0.1)
W2 72 h NP	2.9 (±0.1)	4.0 (±0.1)	1.5 (±0.1)	85.0 (±0.2)	1.1 (±0.1)	17.0 (±0.1)	24.4 (±0.2)	80.2 (±0.5)	87.2 (±1.0)	92.4 (±1.4)	18.6 (±0.1)	0.7 (±0.2)
W2 72 h M	3.1 (±0.1)	4.1 (±0.1)	1.4 (±0.1)	88.2 (±0.1)	1.3 (±0.1)	15.1 (±0.2)	19.1 (±0.4)	78.3 (±0.2)	90.2 (±1.0)	95.0 (±0.3)	14.2 (±0.1)	0.6 (±0.1)

This table highlights the impact of fermentation on gari pH and titratable acidity: pH is slightly lower and titratable acidity is higher as a result of lactic bacteria action. Operator 1 provides a more constant starch content than operator 2. The lightness (L*) of his gari also remains high, and its b* values are overall lower than for operator 2’s gari when the conventional dry matter conditions of the fermented mash to be roasted are no longer used. Similarly, operator 1 manages to keep fairly similar grain sizes regardless of the stipulated fermented mash dry matter content. Conversely, operator 2 produces a generally more gelatinised gari.

As regards the cyanide potential, neither operator’s gari complies with the expectations of the regulatory authorities (safe levels in cassava food products being 10 ppm; Nambisan, 2011). However, operator 2, by virtue of his roasting conditions, produces lower cyanide potentials (dry matter basis), when roasting non‐conventional fermented mash.

Operator 1 produces a gari with sensory properties (colour, particle size) more highly rated by the operators (local survey), but his roasting conditions lead to a smaller reduction in cyanide potential. Operator 1 adjusts the quality of gari based on the criteria to which he has access (size, colour). Conversely, in terms of the responses to which he does not have access, such as cyanide content or gelatinisation rate, he produces a less satisfactory gari.

### Statistical analyses

Table 3 presents the correlation matrix of physico‐chemical quality attributes of the different garis produced by operators 1 and 2.

**Table 3:** Pearson Correlation matrix of physico‐chemical quality attributes of the different garis produced by operators 1 and 2

Variables	**Swelling capacity (/)**	**pH (/)**	**Titratable acidity (%)**	**Colour**			**Total dietary fibre (mg/100g d.b.)**	**Starch content (%)**	**Particle size d_50_ (mm)**	**Starch gelatinisation (%)**	**Dry matter (%)**	**Cyanide potential (mg/100 g)**
				L* **(/)**	a* **(/)**	b* **(/)**						
Swelling capacity (/)	**1**	−0.094	−0.053	**0.616**	−0.569	−0.489	−0.241	0.340	−**0.792**	−0.243	−0.263	0.117
pH (/)	−0.094	**1**	−**0.844**	0.003	−0.334	−0.029	**0.601**	−0.018	0.257	−0.187	0.213	−0.197
Titratable acidity (%)	−0.053	−**0.844**	**1**	−0.256	0.530	0.265	−0.243	−0.256	0.092	−0.022	−0.045	0.500
L* (/)	**0.616**	0.003	−0.256	**1**	−**0.812**	−**0.933**	−0.351	**0.846**	−**0.844**	−**0.584**	−0.274	−0.130
a* (/)	−0.569	−0.334	0.530	−**0.812**	**1**	**0.760**	0.055	−**0.700**	**0.678**	0.479	0.336	0.095
b* (/)	−0.489	−0.029	0.265	−**0.933**	**0.760**	**1**	0.221	−**0.912**	**0.720**	**0.601**	0.436	0.076
Total dietary fibre (mg/100 g d.b.)	−0.241	**0.601**	−0.243	−0.351	0.055	0.221	**1**	−0.185	0.543	−0.331	0.302	0.352
Starch content (%)	0.340	−0.018	−0.256	**0.846**	−**0.700**	−**0.912**	−0.185	**1**	−**0.725**	−0.518	−0.361	−0.094
Particle size d_50_ (mm)	−**0.792**	0.257	0.092	−**0.844**	**0.678**	**0.720**	0.543	−**0.725**	**1**	0.235	0.341	0.089
Starch gelatinisation (%)	−0.243	−0.187	−0.022	−**0.584**	0.479	**0.601**	−0.331	−0.518	0.235	**1**	−0.024	−0.428
Dry matter (%)	−0.263	0.213	−0.045	−0.274	0.336	0.436	0.302	−0.361	0.341	−0.024	**1**	0.057
Cyanide potential (µgHCN g^−1^ dry matter)	0.117	−0.197	0.500	−0.130	0.095	0.076	0.352	−0.094	0.089	−0.428	0.057	**1**

The values in bold are different from 0 to significance level alpha = 0.05.

The objective of this first statistical study is to show that the implemented approach produced roasted garis with different physico‐chemical properties.

**Figure 2:**
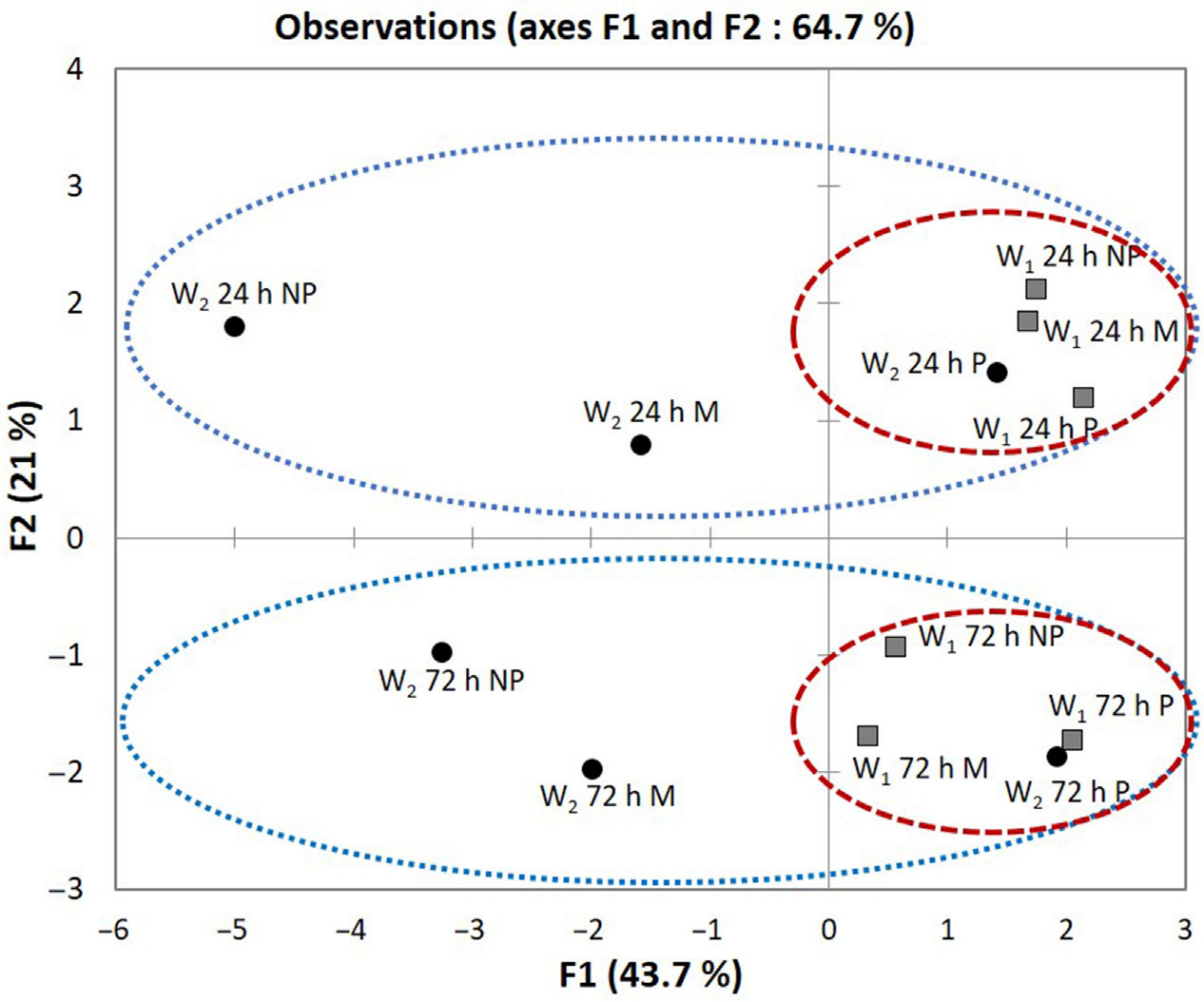
Projection of the 12 garis onto components F1 and F2 of the principal component analysis (PCA) performed on physico‐chemical quality attributes of the 12 garis produced by operator 1 (W1) and operator 2 (W2). The physico‐chemical attributes being CN: cyanide potential (mg/100 g), TDF: total dietary fibre (mg/100 g d.b), TA: titratable acidity (%), DM: dry matter (%) d50: particle size d50 (mm), SG: starch gelatinization (%), pH: hydrogen potential (/),L*: black to white lightness (/),a*: red to green transition (/),b*: blue shading (/), STC: starch content (%), SWC: swelling capacity (/). [Colour figure can be viewed at wileyonlinelibrary.com]

Projection of the 12 garis onto components F1 and F2 of the PCA discriminates the 24 h and 72 h fermented products on axis F2, regardless of the operator and the fermented mash dry matter (Fig. 2).

This projection also reveals that for all conditions studied (P, NP, M), operator 1 managed to adapt process parameters in order to keep all his products in the same group for a given fermentation, whereas the process parameters adopted by operator 2 led to garis with high variability. However, it is important to note that under conventional pressing conditions (24 h P and 72 h P), the garis roasted by operator 2 are in the same group as the garis roasted by operator 1, for each fermentation.

These results reveal the importance of process parameters during roasting operation. It emerges that process parameters adopted by the operator 1 allow to compensate the variability of the mash water content during roasting.

**Figure 3:**
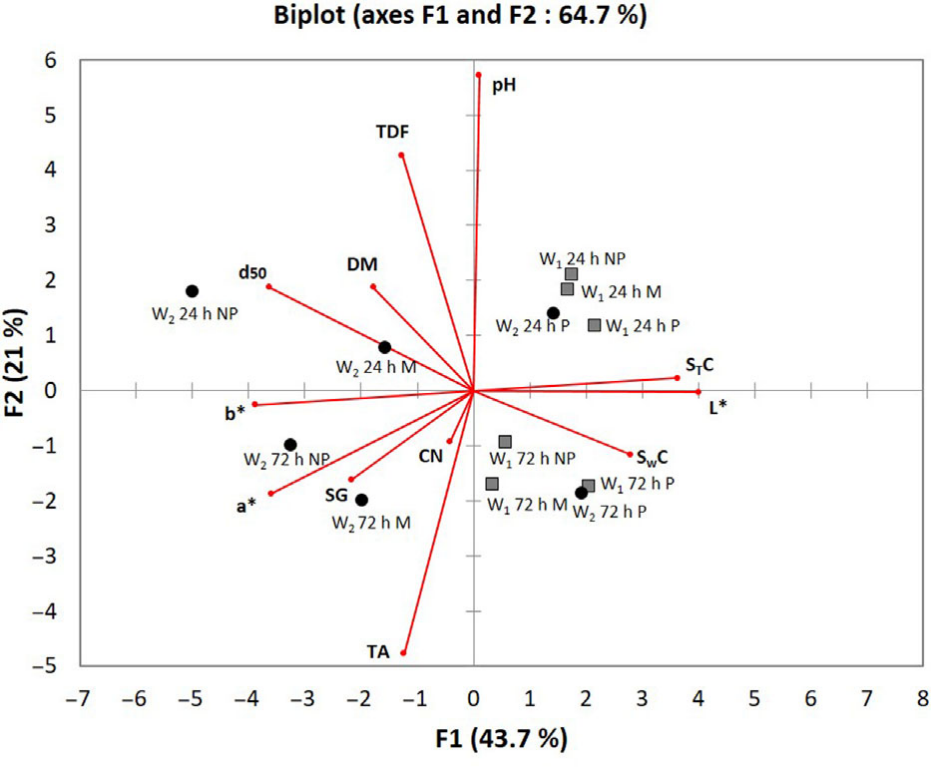
Projection of the physico‐chemical quality attributes (variables) and the 12 garis produced by operator 1 (W1) and operator 2 (W2) onto components F1 and F2 of the principal component analysis (PCA). CN: cyanide potential (mg/100 g), TDF: total dietary fibre (mg/100 g d.b), TA: titratable acidity (%), DM: dry matter (%) d50: particle size d50 (mm), SG: starch gelatinization (%), pH: hydrogen potential (/),L*: black to white lightness (/),a*: red to green transition (/),b*: blue shading (/), STC: starch content (%), SWC: swelling capacity (/).

The explanatory variables – titratable acidity and pH – which make up axis F2 confirm the discrimination of the fermented products on this axis (Fig. 3). This observation is consistent with the action of fermentation which increases organic acids content, especially lactic acid (Brauman *et al*., 1996; Escobar *et al*., 2018), leading to a fall in pH and an increase in titratable acidity.

The projections of the garis produced by operator 1 on axis F1 highlight the characteristics common to these garis (Fig. 3): high lightness (L*) and starch content values, and low d_50_, a* and b* values which distinguish them from operator 2’s garis. The roasting parameters adopted in the case of operator 1 helped preventing browning of the gari, maintained the starch content and produced a gari with the grain size sought by local consumers (local survey).

As regards operator 2’s garis, they present highly variable quality (projection scatter on axes F1, F2, Fig. 3), distributed in all four quadrants. With the exception of garis manufactured by operator 2 under conventional pressing conditions, they are characterised by higher d_50_ values, low lightness (L*) and swelling capacity values and starch contents, and high a* and b* values. So these garis are coarser, browner in colour and swell less in the presence of water. In addition, they are less rich in starch, which has been partly lost due to product sticking to the wok (video observations). Whether for variables associated with their colour, their size or their starch content, the results show that the adopted process parameters by operator 2 for unconventional mash in terms of dry matter content (pressing) are not optimal. In situ surveys confirmed that consumers did not appreciate the quality of these garis. In the case of fermented mash obtained from conventional pressing, process parameters adopted by the operator 2 led to a light and less coarse gari, with a higher starch content, comparable to those produced by the operator 1 and highly rated in terms of quality (local survey).

**Figure 4:**
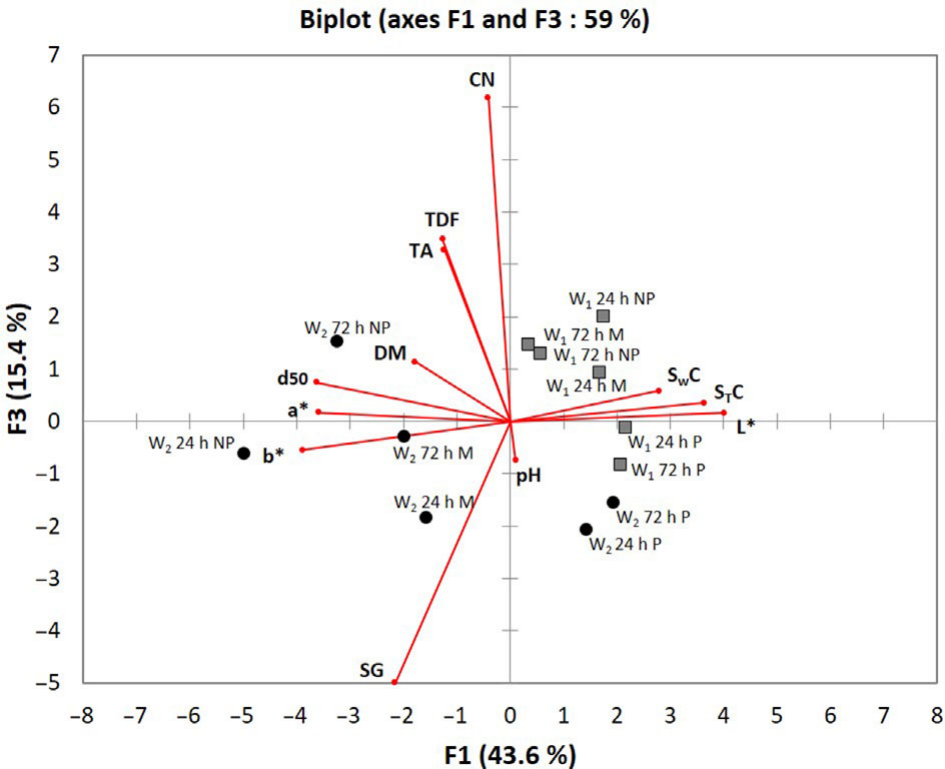
Projection of the physico‐chemical quality attributes (variables) and the 12 garis produced by operator 1 (W1) and operator 2 (W2) onto components F1 and F3 of the principal component analysis (PCA). CN: cyanide potential (mg/100 g), TDF: total dietary fibre (mg/100 g d.b), TA: titratable acidity (%), DM: dry matter (%) d50: particle size d50 (mm), SG: starch gelatinization (%), pH: hydrogen potential (/),L*: black to white lightness (/),a*: red to green transition (/),b*: blue shading (/), STC: starch content (%), SWC: swelling capacity (/).

The projections on axes F1, F3 reveal that operator 2 made garis with a lower cyanide potential and higher starch gelatinisation rate than operator 1 (Fig. 4). This is in accordance with the more marked brown colour, which reflects greater roasting, promoting starch gelatinisation and elimination of HCN. Although the garis made by operator 2 seem in theory not to fully meet consumer expectations based on sensory criteria, it is important to emphasise that in terms of physico‐chemical data, operator 2’s garis are a bit closer to safety standards.

All these results reveal the importance of roasting parameters and indirectly of the behaviour and actions of the operators. Starting with similar mash for roasting, operators produced garis with different qualities. In order to further characterise the impact of roasting process parameters, an analysis of the operators’ behaviour and actions was conducted during the roasting of various mashes with varying water contents.

### Analysis of the process parameters adopted by the operators during the roasting operation

**Figure 5:**
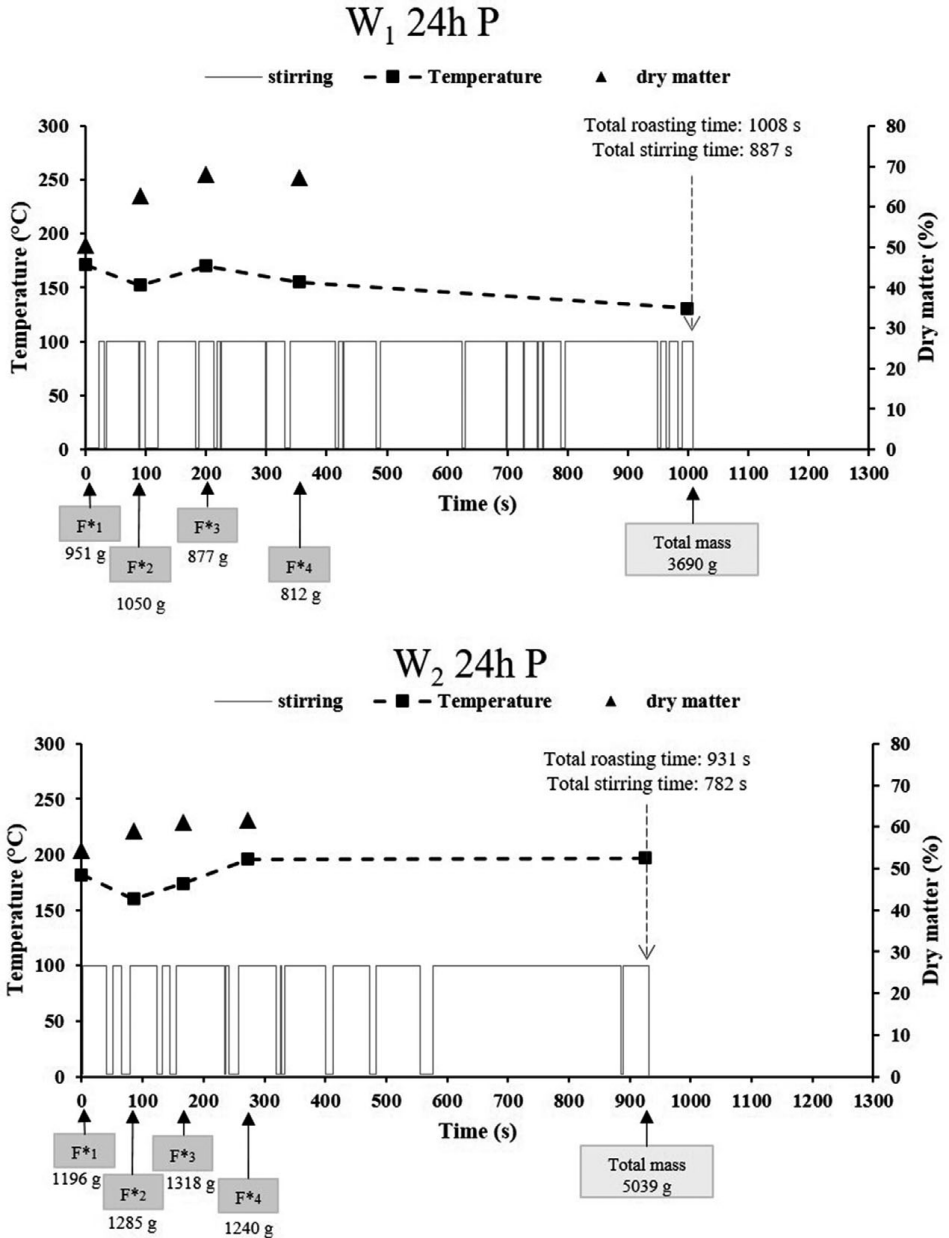
Frequency and duration of stirring and temperature choices adopted by operators 1 (W1 24 h P) and 2 (W2 24 h P) during the roasting of the 24 h fermented pressed mash. The dry matter of mash in the wok just before additions, the time and the number of mash additions during cooking as well as their specific mass (F*x) are also given.

**Figure 6:**
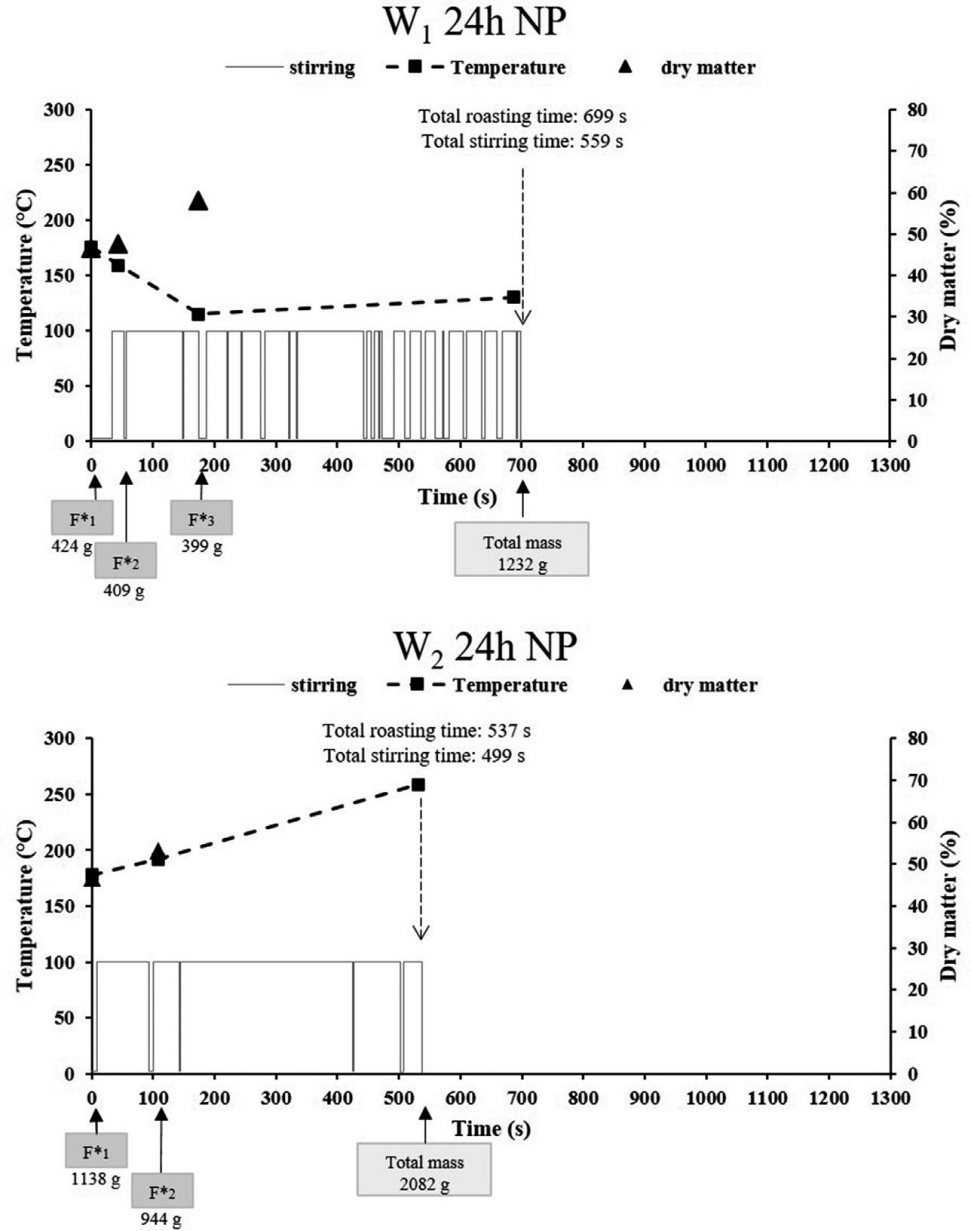
Frequency and duration of stirring and temperature choices adopted by operators 1 (W1 24 h NP) and 2 (W2 24 h NP) during the roasting of the 24 h fermented non‐pressed mash. The dry matter of mash in the wok just before additions, the time and the number of mash additions during cooking as well as their specific mass (F*x) are also given.

**Figure 7:**
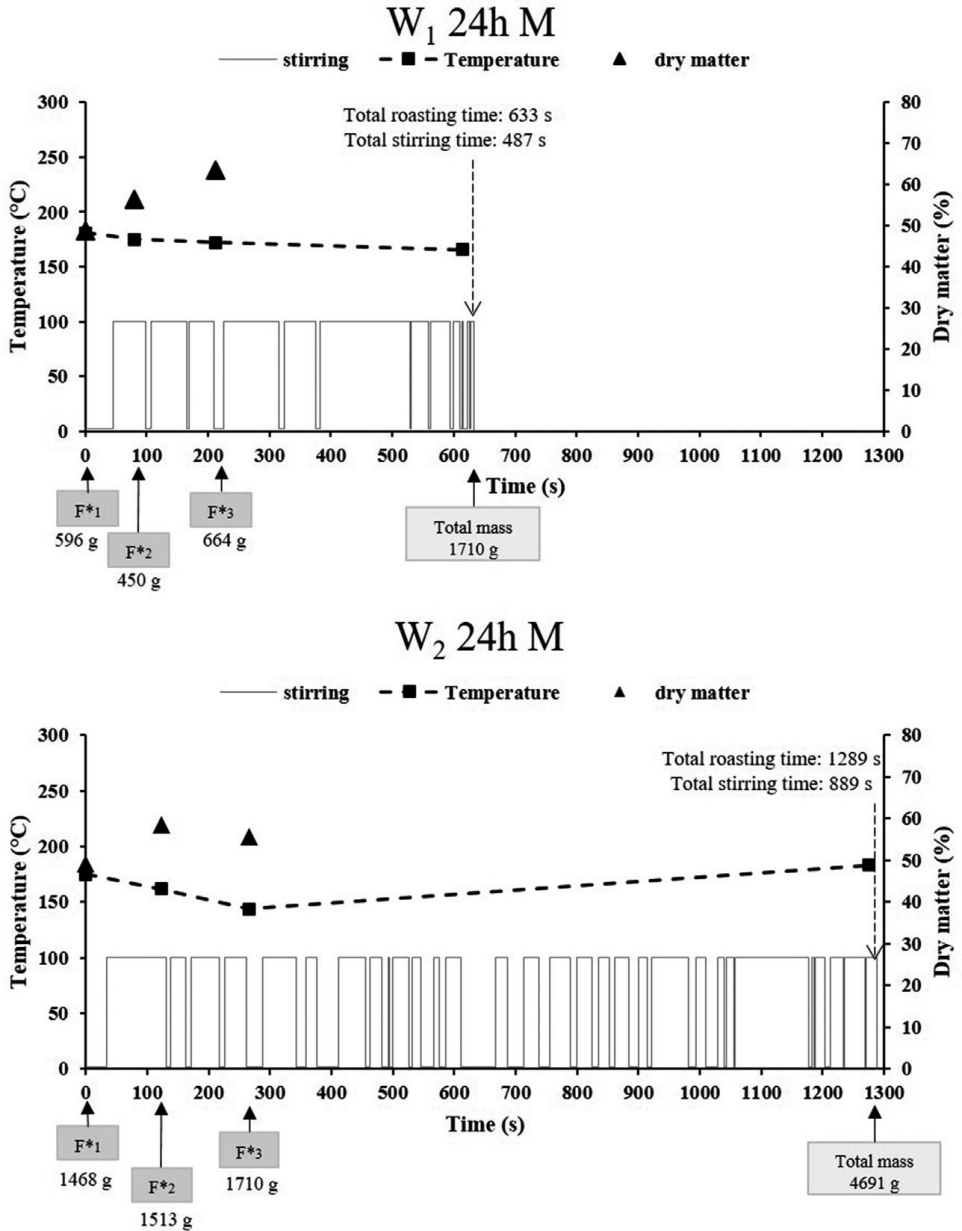
Frequency and duration of stirring and temperature choices adopted by operators 1 (W1 24 h M) and 2 (W2 24 h M) during the roasting of the 24 h fermented mixed mash. The dry matter of mash in the wok just before additions, the time and the number of mash additions during cooking as well as their specific mass (F*x) are also given.

The process parameters adopted by the operators, during roasting of the 24 h fermented pressed, non‐pressed and mixed mashes, are presented in Figs 5, 6 And 7.

The duration of the various stirring cycles is presented for each roasting operation in the lower part of each graph, where a stirring action is represented by the value 100 and non‐stirring by a zero value. For each roasting operation, the total roasting time and total stirring time are also indicated.

Among the six measurements adopted to describe process parameters, four of them were associated with the fermented mash supply mode chosen by the operator, that is number of mash additions, masses of the additions (F*x), individual roasting time for each addition and the fermented mash quantity for roasting. These four data items are presented on the time axis. Two other parameters were measured such as temperature at the centre of the wok upon the additions and the dry matter content of the roasting product present in the wok just before the addition. These data are presented via two curves on the graph.

All of these parameters were analysed for each operator and each roasting scenario.

#### Roasting the 24 h fermented pressed mash (conventional water content)

Analysis of the graphs (Fig. 5) shows the same number of additions for the two operators (four additions), with greater additions for operator 2. In fact, operator 1 added smaller quantities of product (between 812 g and 1050 g), with longer time intervals between additions (between 81 s and 154 s). Upon the addition, the roasting product dry matter content was found to be between 62% and 67%. The temperatures upon the additions varied between 152 and 171°C, reaching 131°C at the end of roasting, with a total roasting time of 1008 s.

As regards operator 2, the quantities of product added during roasting varied between 1196 g and 1318 g, with time intervals between additions of 82 to 106 s. Operator 2 decided to make additions at lower dry matter content (between 59 and 61 %) and adopted higher roasting temperatures (temperatures upon the additions of between 160 and 196°C, and temperature at the end of roasting of 197°C), as compared to operator 1. His roasting choices were consistent with a shorter total roasting time (931 s).

Analysis of the stirring cycles shows that the stirring time/total roasting time ratios for the two operators are fairly similar (88% and 84%, for operator 1 and operator 2, respectively). However these cycles were differentiated by their duration and number: operator 1 preferred to alternate stirring and non‐stirring actions (more stirring cycles, but shorter ones), whereas operator 2 adopted longer stirring cycles. These differences were even more marked at the end of roasting (from 600 s).

The differences in process parameters analysed above had little impact on the end quality of the gari. The garis produced by the two operators under these conditions were within the same group of garis, presenting fairly similar qualities according to the PCA.

#### Roasting the 24 h fermented non‐pressed mash (unconventional water content)

For this type of mash (Fig. 6), the two operators adopted different number of additions – higher for operator 1 (three additions) than for operator 2 (two additions). With this non‐pressed mash, the two operators adopted a common approach, reducing the number of additions (two and three additions instead of four). Operator 2 maintained a high added mass, whereas operator 1 adapted the mass of these additions, with a reduction of more than 50 % compared to the conventional pressed product. In addition, this operator also adapted the time intervals between additions, with a reduction (between 44 s and 130 s), whereas operator 2 maintained a similar time interval as for the pressed product. As regards the temperature profiles, operator 1 maintained the same temperature ranges as for the pressed product. Conversely, the temperature profile adopted by operator 2 highlights his choice to constantly increase the wok temperature (to achieve a final temperature of 259°C). This enabled him to ensure a sufficient thermal energy input to roast the non‐pressed mash (high water content) introduced in high quantities. For both operators, the total roasting times were curiously shorter, despite the fermented mash with higher water content.

The analysis of the stirring cycles shows that operator 1 stirred the product 80 % of the roasting time, whereas operator 2 stirred it 93% of the roasting time. These cycles are very distinctly differentiated by their duration and number. Operator 1 preferred to alternate the stirring and non‐stirring actions, with an even higher number of stirring cycles than with the pressed product, but shorter. Operator 2 only interrupted his stirring four times.

For roasting a non‐pressed mash, operator 2 was more dynamic in his actions than operator 1. This heavy stirring can be explained by his parameter choices during roasting, that is (i) by the limited number of additions (only 2); (ii) by average addition of masses greater than those of operator 1 (more than double); (iii) by higher temperatures, causing sticking of the product, and forcing him to stir practically constantly to unstick it. Moreover, upon his last addition, the gari already present a lower dry matter content than when operator 1 made his last addition. All these factors show that operator 2 was left with a large quantity of material to roast in his wok, with high water content, and also a high roasting temperature; this promoted sticking, agglomeration and browning of the product. The dynamic actions of operator 2 were not sufficient to offset the high water content of this product (a very different gari from the others according to the PCA). For this type of non‐pressed mash, operator 1 adapted his roasting parameters by reducing the mass of additions and slightly reducing the average roasting temperature. These changes seemed sufficient to ensure a better gari quality than the one made by operator 2 (lower d_50_ value, lighter, less brown colour, higher swelling capacity and higher starch content and dry matter content). This gari belongs to the group of garis obtained from the pressed mash. It is interesting to notice that in terms of the two parameters that this operator cannot measure, that is cyanide potential and starch gelatinisation rate, his product deviated from the conventional product (24 h fermented pressed mash).

#### Roasting the 24 h fermented mixed product

For this mash of intermediate water content (Fig. 7), both operators also reduced their number of additions compared to the mash with conventional water content (three additions instead of four for both operators). Operator 2 applied higher addition masses, while operator 1 added masses of the same order of magnitude as for the non‐pressed mash. The temperature profile of the wok for operator 1 highlighted for the first time his choice to maintain a higher temperature, reaching a final temperature of 181°C, and leading him to cut short the total roasting time (633 s).

Operator 2 greatly reduced the average roasting temperature (between 144°C and 175°C, and 183°C at the end of roasting) compared to that used for the first two mashes. This choice led him to considerably extend the total cooking time (1289 s).

The stirring cycle analysis shows that both operators reduced stirring times compared to the pressed product. Solely in this case, the stirring adopted by operator 1 was distinguished by a higher stirring time/total roasting time ratio than that adopted by operator 2 (77% and 69%, respectively). When roasting a mixed mash, operator 2 adapted his actions, reducing the stirring cycle duration while increasing the number. These cycles were very distinctly different from the cycles adopted by the same operator with a pressed or non‐pressed mash. However, these changes in process conditions were not sufficient to ensure a gari of quality comparable to that of operator 1. The gari obtained by operator 2 from the mixed mash was distinct on the PCA projection compared to the group of garis obtained from the pressed mash, while operator 1 managed to keep all these garis in the same group whatever the water content of the fermented mash.

## Conclusions

The objective of this work was to study the impact of process parameters adopted by the operators during the roasting operation of various mashes with varying water content, on gari quality. The experimental approach consisted in selecting two experienced operators and in roasting various mashes with either a conventional or conversely a non‐optimal water content.

Results confirmed that gari quality is largely based on the process parameters adopted by the operators. Indeed, one of the operator ensured a similar quality of all his garis whatever the water contents of the mash. To do so, this operator greatly reduced the addition masses, the total roasting time and adapted the roasting temperature. Few differences could be observed in his actions. This operator succeeded through his choices of roasting parameters to maintain garis of similar qualities, regardless of the water content of the mash for roasting.

The second operator modified his choices in term of masses and number of additions, total roasting time, temperatures and, greatly, number and duration of stirring cycles) observed for the different mash water contents, the quality of the garis obtained from non‐conventional mash remained very different from that of garis obtained from a conventional mash.

This study shows that technological improvements to the gari process can be achieved by appropriate management of the roasting process. However, controlling the water content of the mash before roasting can contribute substantially to gari quality, and the role of process parameters is diminished.

Moreover, cyanide content was too high in all the garis produced showing that it is now essential to raise operator awareness of this major health risk, so as to help them adapt their roasting method. To achieve a goal of optimising operating conditions in the gari roasting process, further experiments are needed to test different combinations of stirring conditions, roasting temperature and duration, mash additions, mash water content, etc. to identify the optimal conditions allowing to produce gari complying with sanitary recommendations and sensory characteristics.

## Ethics approval

Ethics approval was not required for this research.

## Author Contribution

**Layal Dahdouh:** Conceptualization (equal); Data curation (lead); Formal analysis (equal); Investigation (equal); Methodology (equal); Software (lead); Validation (equal); Visualization (equal); Writing‐original draft (lead); Writing‐review & editing (equal). **Andrès Escobar:** Data curation (equal); Formal analysis (equal); Methodology (equal); Software (equal); Writing‐original draft (equal). **Eric Rondet:** Conceptualization (equal); Data curation (equal); Formal analysis (equal); Investigation (equal); Methodology (equal); Supervision (equal); Validation (equal); Writing‐review & editing (equal). **Julien Ricci:** Data curation (equal); Formal analysis (equal); Investigation (equal); Methodology (equal); Validation (equal). **Genevieve Fliedel:** Conceptualization (equal); Funding acquisition (equal); Project administration (equal); Validation (equal). **Laurent Adinsi:** Data curation (equal); Formal analysis (equal); Investigation (equal); Methodology (equal). **Dominique Dufour:** Conceptualization (equal); Funding acquisition (equal); Project administration (equal); Supervision (equal); Validation (equal); Writing‐review & editing (equal). **Bernard Cuq:** Supervision (equal); Validation (equal). **Michele Delalonde:** Conceptualization (equal); Data curation (equal); Methodology (equal); Software (equal); Supervision (equal); Validation (equal); Visualization (equal); Writing‐original draft (equal); Writing‐review & editing (equal).

### Peer review

The peer review history for this article is available at https://publons.com/publon/10.1111/ijfs.14745.

## Data Availability

The data that support the findings of this study are available from the corresponding author upon reasonable request.
